# Bone Regulates Glucose Metabolism as an Endocrine Organ through Osteocalcin

**DOI:** 10.1155/2015/967673

**Published:** 2015-03-19

**Authors:** Jin Shao, Zhi Wang, Tieyi Yang, Hui Ying, Yan Zhang, Shuyi Liu

**Affiliations:** Department of Orthopaedics, Shanghai Pudong New Area Gongli Hospital/Clinical School, The Second Military Medical University, Shanghai 200135, China

## Abstract

Skeleton was considered as a dynamic connective tissue, which was essential for mobility, calcium homeostasis, and hematopoietic niche. However more and more evidences indicate that skeleton works not only as a structural scaffold but also as an endocrine organ, which regulates several metabolic processes. Besides osteoprotegerin (OPG), sclerostin (SOST), and Dickopf (DKK) which play essential roles in bone formation, modelling, remodelling, and homeostasis, bone can also secret hormones, such as osteocalcin (OCN), which promotes proliferation of *β* cells, insulin secretion, and insulin sensitivity. Additionally OCN can also regulate the fat cells and male gonad endocrine activity and be regulated by insulin and the neural system. In summary, skeleton has endocrine function via OCN and plays an important role in energy metabolism, especially in glucose metabolism.

## 1. Introduction

Bone has been only considered as a structure organ for centuries. Classical understanding of skeleton is that bone functions as the structure scaffold in animals and contains three types of cells: osteoblast, osteocyte, and osteoclast. Osteoblasts come from mesenchymal stem cells and can differentiate to osteocytes, which stay in the bone matrix. Osteoblasts are responsible for bone formation, and its activity can be regulated by several pathways such as WNT signaling pathway. Osteoclasts are a type of bone cells, which resorb bone and derived from the fusion of mononuclear cells belonging to the monocyte/macrophage lineage. Osteocytes and osteoblasts can secrete some signaling factors, such as WNT, OPG, and SOST, in order to regulate the coupling function of osteoblast and osteoclast; in this way bone obtains the ability to model and remodel itself. Recent reports indicate that SOST, a WNT antagonist, downregulates the WNT signaling activity by crosstalk with parathyroid hormone (PTH) and bone morphogenetic protein (BMP) in order to regulate bone cell differentiation, proliferation, bone formation, and bone resorption activities [1–4]. Even though there are plenty of evidences indicating that bone can secrete protein factors in the circulation and regulate itself, all these secreted factors cannot be called hormones.

**Figure 1:**
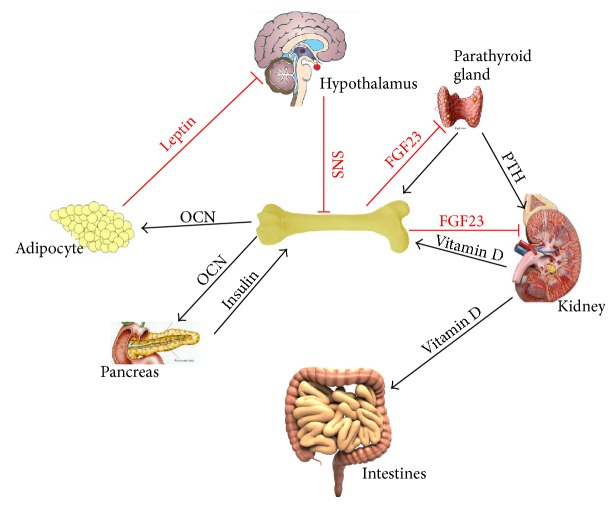
Skeleton regulates mineral and energy homeostasis. In mineral homeostasis, low level of circulating calcium stimulates the parathyroid gland to release PTH, which later upregulates blood calcium levels by stimulating osteoclastic bone resorption, renal calcium reabsorption, and renal production of Vitamin D to increase intestinal calcium absorption. Increased serum phosphate and Vitamin D stimulate FGF23 synthesis and releasing in bone, which subsequently inhibits PTH production from the parathyroid gland, inhibits Vitamin D production in the kidney, and promotes renal phosphate excretion. Leptin inhibits bone formation and the homeostatic function of the skeleton indirectly through SNS. However, SNS signalling also increases the production of OCN from bone, which feeds into the positive loop. OCN affects pancreatic *β* cells and increases insulin level, which has feedback effect on bone, driving further production of OCN. OCN also acts on fat to increase the production of adiponectin and upregulates insulin sensitivity. FGF23, fibroblast growth factor 23; PTH, parathyroid hormone; SNS, sympathetic nervous system.

However, in the past decades with the development of technology we found that skeleton can also be an endocrine organ, which regulates not only itself but also other organs. The most well known and studied two hormones secreted by skeleton are OCN and fibroblast growth factor 23 (FGF23) [5] (Figure 1). In this review we would focus on OCN, especially on its function as a hormone in metabolism homeostasis.

## 2. Endocrine Functions of Bone through OCN

### 2.1. Production of OCN

OCN is specially synthesized and secreted by cells of osteoblast lineage, such as osteoblast and osteocyte [6, 7]. In humans OCN is also known as bone *γ*-carboxyglutamic acid-containing protein (BGP) and it is one of the richest noncollagenous proteins in bone matrix. The most of OCN is found in bone matrix and only a small amount in blood, because it has strong affinity to bone matrix.

**Figure 2:**
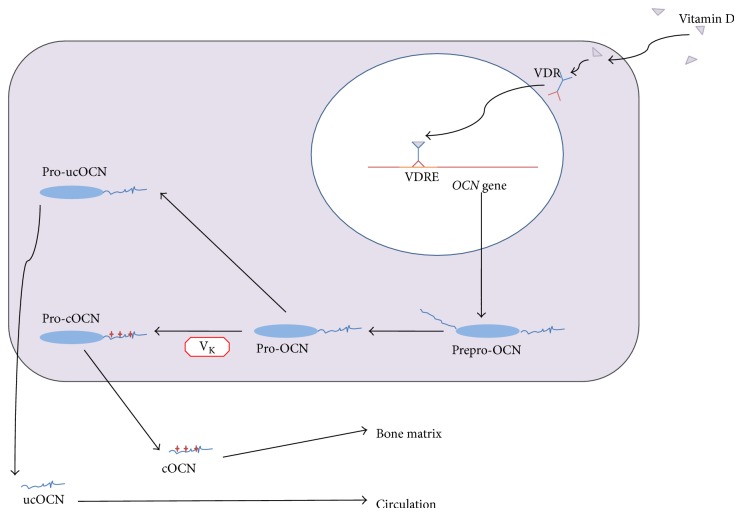
OCN is synthesized in osteoblasts.* OCN* is mainly expressed in osteoblasts. After transcription, which is stimulated by Vitamin D, the prepro-OCN peptide will be proteolysed and forms a prepeptide (23 aa) and a pro-OCN peptide (75 aa). The pro-OCN peptide can be carboxylated at Glu residues 17, 21, and 24, resulting in formation of Gla residues in a Vitamin K dependent process. Generally, this process only occurs in a proportion of newly synthesized proosteocalcin. Then Gla and Glu pro-OCN peptides are subjected to a final proteolytic process that produces cOCN and ucOCN. Both forms are released from osteoblasts in a process which is calcium dependent. While the carboxylated Gla residues are involved in calcium and hydroxyapatite binding, allowing OCN deposition on mineralized bone matrix, ucOCN has a low affinity for hydroxyapatite and is more easily released into the circulation. OCN, osteocalcin; ucOCN, uncarboxylated osteocalcin; VDR, Vitamin D receptor; VDRE, Vitamin D receptor element.

OCN has proosteoblastic or bone-building function. Osteoblasts can secret OCN to stimulate osteoblastic differentiation and osteocytic maturation. The* OCN* gene has 4 exons and 3 introns, and more than 70% of the exon sequences are conserved both in human and in mice. The OCN protein contains 46–50 amino acid residues (varying from different species), the synthesis process of which is a little complicated (Figure 2). Firstly, the transcription is regulated by 1*α*,25-dihydroxy-Vitamin D3. After the translation it is only preproosteocalcin, which contains 98 amino acid residues composed of three parts: a 23-residue signal protein, a 26-residue target propeptide, and a 49-residue mature protein. Proteolysis of the prepropeptide will form the mature OCN [8]. At position 17, 21 and 24 of the 49-residue peptide second carboxyl groups (–COOH) are added to form *γ*-carboxyglutamyl residues, which are essential for its activity regulation.

According to the carboxylation level, OCN can be divided into two groups; one is called undercarboxylated osteocalcin (ucOCN), and the other is carboxylated osteocalcin (cOCN). The decarboxylated form ucOCN is the active form of OCN as a hormone, which has little affinity to bone so that most of ucOCN will circulate with the blood, while most of cOCN will be stored in the bone matrix because of its strong affinity to bone matrix. Usually the concentration of ucOCN is not controlled by the protein synthesis, but by the decarboxylation of OCN and its releasing from the bone matrix. This issue will be discussed in Section 2.3 later.

### 2.2. OCN Targeting Organs

#### 2.2.1. OCN Targets *β* Cells and Adipocytes

There are evidences indicating that in* Ocn* knocked out mice the total amount of insulin in serum is downregulated, and the* Ocn* knocked out mice have so-called impaired glucose-stimulated insulin secretion (GSIS) and poor glucose tolerance phenotype [9]. At the same time the serum adiponectin, a protein hormone that modulates several metabolic processes such as glucose regulation and fatty acid oxidation, was also reduced [10]. All these results suggest that OCN may target *β* cells and insulin targeting tissue such as muscle, liver, and adipocyte in order to regulate both insulin secretion and sensitivity [11, 12]. Later studies show that the change of insulin sensitivity is probably mediated by adiponectin instead of direct interaction with OCN [13]. This result indicates that adipocytes are probably one target of ucOCN, and ucOCN functions as the activity form of OCN in circulation.

There are also many evidences indicating that ucOCN can regulate *β* cells in pancreatic islets. Not only insulin 1 and insulin 2 can be upregulated by ucOCN but also the proliferation of *β* cells. Further research indicates that CyclinD1, CyclinD2, and Cdk4 in *β* cells can also be regulated by ucOCN, and in this way it is not difficult to understand why ucOCN can stimulate *β* cell proliferation [9, 12, 14]. *β* cells proliferation and insulin secretion can be significantly affected at low concentrations of ucOCN (6–60 pM) [15]. The mice lacking osteoblasts have not only problems on bone density and strength but also impaired glucose metabolism, such as high blood glucose, low insulin secretion, and insulin resistance. All these phenotypes mimic the defects in* Ocn* knocked out mice. However, administration of OCN can restore glucose and insulin level in circulation but only partially insulin sensitivity [16]. This indicates that osteoblasts may have some OCN independent ways to regulate insulin sensitivity.

Although more and more evidences show that OCN can regulate *β* cells and increase the insulin sensitivity of insulin targeting cells, the receptor of OCN or more correctly ucOCN has not been identified yet. What is more, we do not even know how OCN regulates the insulin sensitivity. There are only evidences indicating adiponectin mediates the insulin sensitivity regulation of OCN [13]. Whether some other mechanisms involved in this progress is still not clear. We should also not ignore cOCN, which usually stays in the bone matrix. The biological function of cOCN is still unclear. Is it only storage of ucOCN? Does it have some unknown functions on metabolism of bone formation? Some* in vitro* studies indicated that cOCN could regulate bone formation and resorption; however, in the* Ocn* knocked out mice no negative bone effects were observed [17–19]. Anyway, a lot of questions still need to be answered.

#### 2.2.2. OCN Regulates Testicular Function

OCN regulates male reproduction activity [20]. These were observed from the experimental studies on* Esp* and* Ocn* knocked out mice. With or without OCN, male mice showed different reproductive activity. Mice with high OCN activity (the* Esp *knocked out mice) had increased testicular volume and sperm count. On the contrary, the OCN absent mice (the* Ocn* knocked out mice) showed shriveled testes, epididymis, and seminal vesicles. However, the female in both* Esp* and* Ocn* knocked out groups did not show any defects in reproduction.* Ocn* specific deletion in leydig cells showed no such effects as described above [20]. This indicates that OCN secreted by skeleton regulates male reproduction activity as a hormone.

**Figure 3:**
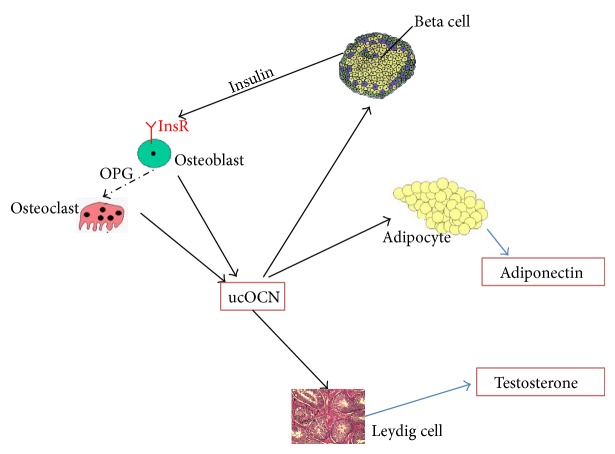
Endocrine actions of osteocalcin. Circulating OCN and particularly ucOCN (released during bone resorption activity) have direct effects on *β* cells, stimulating insulin synthesis. Meanwhile it affects adipocytes and stimulates adiponectin secretion. Adiponectin upregulates insulin sensitivity. In turn, insulin binds to insulin receptor of osteoblasts and affects osteoblasts; then OPG expression will be upregulated and in turn suppress the activity of osteoclasts. Osteoclast stimulates bone resorption with subsequent release of ucOCN in blood circulation. OCN also functions on leydig cells. It can increase their activity and testosterone production. OCN, osteocalcin; ucOCN, uncarboxylated osteocalcin; OPG, osteoprotegerin; InsR, insulin receptor.

And fortunately the receptor in the leydig cell of ucOCN was identified. It is a G protein-coupled receptor (GPCR6A) and localized on the surface of the leydig cell. This 7-transmembrane protein was found because the* GPCR6A* knocked out mice copy the metabolic syndrome of the* Ocn* knocked out mice [21, 22]. In the past decades it was demonstrated that gonadal hormone (estrogen) can regulate bone formation [23, 24], and now we know that bone can also regulate testicle and testosterone secretion. This discovery suggested that there may be a novel mechanism regulating bone formation in some special stage, such as teenage time, in which rapid bone formation is associated with androgen secretion changing. In general, OCN, particularly ucOCN (released during bone resorption) targets directly *β* cells and stimulates insulin synthesis and secretion. At the same time it affects adipocytes and enhances adiponectin secretion. Adiponectin itself upregulates the insulin sensitivity of the insulin targeting cells [13]. Interestingly, OCN also targets leydig cells and regulates testosterone production and male reproductive activity (Figure 3).

### 2.3. Regulation of OCN Production and Activation

#### 2.3.1. OCN Is Partly Regulated by 1*α*,25-Dihydroxyvitamin D3 and Vitamin K

Vitamin D is a kind of fat-soluble secosteroids which plays an essential role in absorption of calcium, phosphorus, iron, zinc, and their metabolism. In humans and mice the most active form of Vitamin D is 1*α*,25-dihydroxyvitamin D3 (Vitamin D3). Recent studies showed that the* Ocn* expression in osteoblasts can be partly stimulated by Vitamin D3 [25]. It has been reported that OCN usually functions to inhibit mineralization. More interestingly fibroblast growth factor 23 (FGF23), another bone secreted hormone, can affect kidney and inhibit Vitamin D synthesis. This loop suggested a feedback loop, in which bone can partly regulate its own hormone secretion [26].

Studies showed that accumulation of osteocalcin in the ECM of human osteoblastic cultures stimulated by 1*α*,25-dihydroxyvitamin D3 is inhibited by warfarin (antagonist of Vitamin K), while Vitamin K2 enhanced the 1*α*,25-dihydroxyvitamin D3 effect [27], and 1*α*,25-dihydroxyvitamin D3 stimulated mineralization was significantly augmented by warfarin [28].

The biological active form of OCN is ucOCN, while the cOCN has a strong affinity to bone matrix and binds tightly to hydroxyapatite, as a result little cOCN could be found in the serum [29]. Vitamin K is a cofactor for the glutamate carboxylase, which is required for carboxylation. In the absence of Vitamin K the serum level of ucOCN would be increased. On the contrary, high level of Vitamin K can reduce ucOCN level, which had been proved by Vitamin K administration in daily diet [30].

#### 2.3.2. OST-PTP, the Product of Receptor-Type Tyrosine-Protein Phosphatase V (*Esp*) Controls OCN Activity

Generally speaking, there are two ways controlling OCN signaling, one is on the transcription level and the other is on the posttranslational level. The product of* Esp* is an osteotesticular protein tyrosine phosphatase (OST-PTP), which is found in embryonic cells, Sertoli cells, and osteoblast [9]. Karsenty et al. found* Esp* knocked out mice have the opposite phenotype compared with the* Ocn* knocked out group (Table 1). The phenotypes of the* Esp *knocked out mice are as follows: hypoglycemia, high glucose-stimulated insulin secretion, glucose tolerance, pancreatic insulin content, and *β* cell proliferation [9].* Esp* deletion (either totally knocked out or selectively knocked out in osteoblasts) resulted in the exact opposite phenotype as found in the* Ocn* knocked out mice. And* Esp* can rescue the phenotype of the high fat diet and gold thioglucose-induced obesity and diabetes, which mimics the phenotype of ucOCN treated mice [9, 12]. All these results let Lee et al. believe that OST-PTP may mediate the inactivation of OCN via *γ*-carboxylation. Indeed lacking one allele of* Ocn* in the* Esp* knocked out mice rescued the metabolic abnormality of the* Esp* absent mice. This result indicates that OST-PTP downregulates the activity of ucOCN [9]. As the total* Ocn* gene expression did not change in* Esp* knocked out mice, Ferron et al. suggested that OST-PTP was involved in the osteoclast-dependent ucOCN formation and releasing [31].

**Figure 4:**
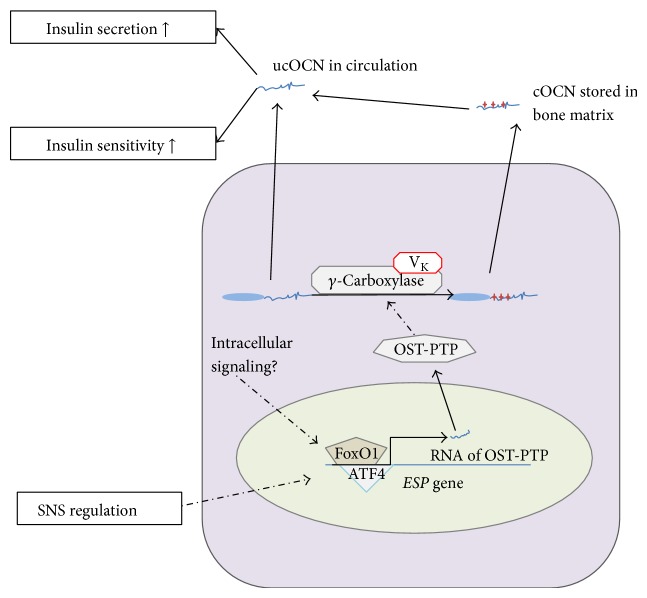
Proposed regulation of* Esp* expression and OCN carboxylation in mice. FoxO1 and ATF4 could bind to the promoter site to stimulate the transcription of* Esp*. The product of* Esp* is OST-PTP, which can suppress the carboxylation of prepro-OCN and in turn suppress the activity of OCN. V_K_, Vitamin K; SNS, sympathetic nervous system; OCN, osteocalcin; ucOCN, uncarboxylated osteocalcin.

In the recent years the molecular mechanism of* Esp* regulation was revealed (Figure 4). Two transcription factors, forkhead box protein O1 (FOXO1) and cyclic AMP-dependent transcription factor (ATF-4), are involved in the regulation of the total OCN [32]. FOXO1 is a conservative modulator of glucose metabolism in many organs, as well as insulin synthesis in *β* cells, and ATF4 regulates expression of many genes which mostly are important for adult bone formation and development [33, 34]. Osteoblast specific* FoxO1* knocked out mice have low blood glucose level and high glucose tolerance [35], which is similar to the mice with high ucOCN level. This indicated that FoxO1 may be a modulator of* Ocn* transcription or posttranslational modification. Further studies on knocked out mice showed that FoxO1 could upregulate OST-PTP. Molecular experimental studies showed that FoxO1 could bind to a cognate FoxO1 binding site in the* Esp* promoter area and stimulate the expression of the downstream* Esp;* then OST-PTP would activate the carboxylation of OCN or ucOCN. The new formed cOCN would bind to hydroxyapatite and stay in bone matrix, and as a result the ucOCN would be downregulated [35]. These studies also found the binding site for ATF4 in the* Esp* promoter. Now it is not surprising that ATF4 can suppress obesity in mice and involve in the glucose homeostasis in human [36–38]. In fact even though ATF4 is mainly expressed in osteoblast, it functions quite similar to FoxO1. Via stimulating OST-PTP transcription ATF4 suppress the activity of OCN, in this way suppressing insulin secretion and insulin sensitivity [39, 40]. More details of OCN regulation are still unknown, and the endocrine regulation of OCN will be discussed in the later section of this review.

## 3. Bone Regulates Glucose Metabolism via OCN

### 3.1. OCN in Glucose Metabolism

As mentioned in the sections above, in the* Ocn* knocked out mice the blood glucose concentration was higher than that of the wild type. Serum insulin in the knocked out mice would be downregulated and the mice tended to be obesity [12]. All these indicate that OCN may be essential in energy metabolism, especially in glucose metabolism. With administration of OCN or deletion of* Esp* (OST-PTP increases ucOCN, the active form of OCN), the mice achieved higher activity of insulin, lower blood glucose, higher glucose tolerance, and higher insulin sensitivity with normal glucagon levels [9]. This abnormality is mainly caused by the increased number or mass of pancreatic islets and *β* cells. This means that OCN can increase insulin secretion and insulin sensitivity in some tissues such as muscles and adipose tissue. The increasing sensitivity of insulin is probably mediated by adiponectin, which can be upregulated by ucOCN [9, 12, 41]. cOCN would be stored in the bone matrix and released during bone resorption [10, 42]. Osteoclasts would secret protons and decrease the pH value during resorption; this would facilitate the decarboxylation of cOCN and by the way the active form ucOCN would enter the circulation to stimulate *β* cells proliferation and insulin secretion [31, 43]. However, more details of the mechanism need to be revealed.

### 3.2. Feed-Forward Effect of Insulin

Insulin, synthesized and modified in *β* cells in pancreas, is an essential protein hormone in almost all vertebrates. Through clinical observation we know that insulin signaling can also affect the bones. Insulin receptor (InsR) is required for osteoblast survival, proliferation, and differentiation [44, 45]. Runx2 is a transcription factor which is essential for osteoblastic differentiation and skeletal morphogenesis. Studies showed that the expression of Runx2 was decreased in osteoblasts lacking InsR [45, 46]. However, insulin dose does not directly regulate Runx2 activity. Instead, insulin downregulates Twist2, an inhibitor of Runx2 activity [46], to suppress the activity of Runx2 [43].

Insulin can also promote bone resorption. However, it does not directly work on osteoclasts but on osteoblasts. Insulin signaling can decrease osteoprotegerin (OPG) expression in osteoblasts via binding to InsR [31]. OPG, a key regulator of osteoclasts, together with receptor activator of nuclear factor-*κ* ligand (RANKL) via receptor activator of nuclear factor-*κ* (RNAK) regulates the differentiation and activity of osteoclasts [2, 47, 48]. During bone resorption the osteoclasts secrete proton and the pH value will be low. This acidic environment facilitates decarboxylation and the concentration of ucOCN will be upregulated. In turn ucOCN increases *β* cells proliferation, insulin secretion, and insulin sensitivity [16, 49]. Thus, insulin signaling and OCN form a feed-forward loop, in which insulin affects osteoblasts and in turn increases its own secretion and sensitivity via ucOCN. Insulin signaling can decrease OPG expression in osteoblast via binding to InsR [31]. And InsR is downstream molecule of OST-PTP, which inhibits phosphorylation of InsR and stops insulin signaling in osteoblasts [50, 51]. This feed-forward model explains why the osteoblastic InsR knocked out mice have low serum insulin level and high insulin resistance.

### 3.3. Endocrine Regulation of OCN

As mentioned above, the relationship between OCN and insulin is not a feedback relationship, so there must be some other signaling pathways to regulate the OST-PTP activity. If not, the serum insulin concentration cannot stay in a certain level with only the feed-forward loop between bone and pancreatic islets. Some studies suggested that sympathetic nervous system (SNS) may control the expression of* Esp* via leptin, a hormone secreted by adipocytes [41]. The main function of leptin is regulating the amount of fat stored in the body. It can adjust both the sensation of hunger and energy expenditures. The target of leptin signaling is the brain. It inactivates the 5-hydroxylase 2 (Tph2), which is known as the rate-limiting enzyme of serotonin synthesis [52].* Tph2* knocked out mice have osteoporotic and anorectic phenotype, indicating this source of serotonin can regulate osteoblasts. Yadav et al. have proved that leptin-dependent regulation of OCN carboxylation appeared to be through the hypothalamus [53, 54]. The SNS acts through *β*2-adrenergic receptors (Adrb2) in osteoblasts to upregulate the* Esp* expression [41], and in so doing the activity of ucOCN would be suppressed.

### 3.4. OCN in Human

#### 3.4.1. *ESP* Does Not Play the Same Role in Human

The* ESP* gene in human is a so-called pseudogene which does not have a functional product. Its function may be replaced by another or some other proteins [55]. Years ago a study showed that in human dephosphorylation of InsR can be achieved by protein-tyrosine phosphatase 1B (PTP1B) instead of OST-PTP [31]. So it seems that the mechanism of OCN regulation in human is different from that in mice.

#### 3.4.2. The Circadian Rhythm of OCN in Human Is Different from That in Mice

In human the concentration of OCN will arrive at its peak in the early morning and its lowest point in the afternoon, while the mice will have a high level of OCN in the day and the level of OCN reaches its lowest point at night during sleep [56]. In the older studies the researchers could only test the concentration of total OCN, now there are several ways to test the level of ucOCN, such as ucOCN specific ELISA and hydroxyapatite (HAP) binding assay [19, 57]. With these technological development, recent studies showed that the ratio of ucOCN/OCN usually did not change, while the total amount of ucOCN in circulation had the similar changes as the circadian rhythm of OCN [8, 58–62].

#### 3.4.3. Clinical Studies of OCN

In clinical studies the data were mostly focused on the effects of OCN on bone formation and metabolism. Children and adolescents, who have the need to increase bone formation, usually have higher levels of OCN and ucOCN and this may facilitate the utilization of glucose in bone [63]. Clinical studies also showed that bone loss was associated with high level of ucOCN in human, and in older people high OCN level predicted increased fracture risk or lower bone density [64, 65]. The phenomenon can be explained by studies in mice that bone resorption facilitates decarboxylation of OCN and releases OCN from bone matrix to the blood [10]. However, whether OCN has effects on skeleton or not is still not clear; the OCN knocked out mice seem quite normal. The minor differences between the knocked out and wild type mice suggest that OCN may play some roles in regulation of bone mineralization [17].

Only in recent years many studies are focused on the hormone functions of ucOCN and OCN in energy metabolism. Patients who suffer from type 1 or type 2 diabetes mellitus have lower level of OCN than healthy people [66–69]. Patients with obesity also show lower level of OCN [70, 71]. We have already discussed that adiponectin mediates the insulin sensitivity. However, studies showed in male patients with type 2 diabetes adiponectin was associated with ucOCN/OCN ratio instead of the concentration of ucOCN, while in female patients it was correlated with OCN, but not ucOCN [72]. It is now very clear that OCN regulates testicular function and does not have such function on female gonad [20]. Whether it has something to do with the differences between male and female diabetic patients is still unknown. Anyway, more studies need to be done to reveal the mechanism of this phenomenon.

## 4. Conclusion

Studies in animal models showed that ucOCN targets *β* cells in the pancreas to directly regulate insulin synthesis and regulates insulin sensitivity through adiponectin [13]. And experiments in mice showed that ucOCN may be a potential therapy for diabetic patients [66]. One of the regulators of OCN is OST-PTP, which promotes the carboxylation of OCN and reduces the concentration of ucOCN or the ratio of ucOCN/OCN [8].

There are also many clinical evidences indicating that OCN or probably ucOCN is associated with fasting glucose and insulin sensitivity [63]. However, OST-PTP is not a functional protein in human, and the exact role which OCN plays in human still needs to be studied.

Anyway all these studies suggest us skeleton can behave not only as a structure scaffold but also as an endocrine organ, which regulates energy metabolism, even though all the studies are mostly done in mice and only partially confirmed in human [55]. Therefore, more investigations are needed to reveal the total functions of OCN as a hormone in energy metabolism.

## Figures and Tables

**Table 1:** Metabolic phenotypes of *ESP* and *Ocn* knocked out mice.

	Δ*ESP* mice	Δ*Ocn* mice
Blood glucose	Decreased	Increased
Glucose tolerant	Increased	Decreased
Sensitive to insulin	Increased	Decreased
Beta cells proliferation	Increased	Decreased
ucOCN	Decreased	Absent
